# Extreme environmental temperatures and motorcycle crashes: a time-series analysis

**DOI:** 10.1007/s11356-022-21151-8

**Published:** 2022-06-06

**Authors:** Mohammad Javad Zare Sakhvidi, Jun Yang, Danial Mohammadi, Hussein FallahZadeh, Amirhooshang Mehrparvar, Mark Stevenson, Xavier Basagaña, Antonio Gasparrini, Payam Dadvand

**Affiliations:** 1 Department of Occupational Health, School of Public Health, Shahid Sadoughi University of Medical Sciences, Yazd, Iran; 2 School of Public Health, Guangzhou Medical University, Guangzhou, 511436 China; 3 Department of Epidemiology and Biostatistics, School of Public Health, Shahid Sadoughi University of Medical Sciences, Yazd, Iran; 4 Department of Occupational Medicine, Faculty of Medicine, Shahid Sadoughi University of Medical Sciences, Yazd, Iran; 5 Melbourne School of Design/Melbourne School of Population and Global Health, The University of Melbourne, Melbourne, Victoria Australia; 6 ISGlobal, Doctor Aiguader 88, 08003 Barcelona, Catalonia Spain; 7 Universitat Pompeu Fabra (UPF), Barcelona, Spain; 8 CIBER Epidemiología y Salud Pública (CIBERESP), Melchor Fernández Almagro, 3-5, 28029, Madrid, Spain; 9 Department of Public Health, Environments and Society, London School Hygiene & Tropical Medicine, London, UK; 10 Centre for Statistical Methodology, London School Hygiene & Tropical Medicine, London, UK; 11 Centre on Climate Change and Planetary Health, London School of Hygiene & Tropical Medicine, London, UK

**Keywords:** Climate change, Time series, Iran, Traffic accident

## Abstract

Extreme temperature could affect traffic crashes by influencing road safety, vehicle performance, and drivers’ behavior and abilities. Studies evaluating the impacts of extreme temperatures on the risk of traffic crashes have mainly overlooked the potential role of vehicle air conditioners. The aim of this study, therefore, was to evaluate the effect of exposure to extreme cold and hot temperatures on seeking medical attention due to motorcycle crashes. The study was conducted in Iran by using medical attendance for motorcycle crashes from March 2011 to June 2017. Data on daily minimum, mean and maximum temperature (°C), relative humidity (%), wind velocity (km/h), and precipitation (mm/day) were collected. We developed semi-parametric generalized additive models following a quasi-Poisson distribution with the distributed nonlinear lag model to estimate the immediate and lagged associations (reported as relative risk [RR], and 95% confidence interval [CI]). Between March 2011 and June 2017, 36,079 medical attendances due to motorcycle road traffic crashes were recorded (15.8 ± 5.92 victims per day). In this time period, the recorded temperature ranged from −11.2 to 45.4 °C (average: 25.5 ± 11.0 °C). We found an increased risk of medical attendance for motorcycle crashes (based on maximum daily temperature) at both extremely cold (1st percentile) and hot (99th percentile) temperatures and also hot (75th percentile) temperatures, mainly during lags 0 to 3 days (e.g., RR: 1.12 [95% CI: 1.05: 1.20]; RR: 1.08 [95% CI: 1.01: 1.16]; RR: 1.20 [95% CI: 1.09: 1.32] at lag0 for extremely cold, hot, and extremely hot conditions, respectively). The risk estimates for extremely hot temperatures were larger than hot and extremely cold temperatures. We estimated that 11.01% (95% CI: 7.77:14.06) of the medical attendance for motorcycle crashes is estimated to be attributable to non-optimal temperature (using mean temperature as exposure variable). Our findings have important public health messaging, given the considerable burden associated with road traffic injury, particularly in low- and middle-income countries.

**Supplementary Information:**

The online version contains supplementary material available at 10.1007/s11356-022-21151-8.

## Introduction

Global climate change and the anticipation of an increase in the frequency, severity, and length of extreme weather conditions worldwide have raised concerns about the impacts of environmental temperature on human health (Vicedo-Cabrera et al. 2021; Yang et al. 2021; Zhao et al. 2021). Associations between extreme environmental temperatures and health outcomes have been well documented (Gao et al. 2016; Gasparrini et al. 2015). An emerging body of evidence has also linked extreme environmental temperatures and other meteorological conditions with an elevated risk of unintentional injuries such as road and occupational injuries (Gariazzo et al. 2021; im Kampe et al. 2016; Liang et al. 2022; Marinaccio et al. 2019; Martínez-Solanas et al. 2018; Xing et al. 2019). Specifically, extreme environmental temperatures and precipitation events have been associated with an increased risk of road traffic crashes (Basagaña et al. 2015; Lee et al. 2014; Liu et al. 2017). Such associations are likely mediated through the effects of these meteorological conditions on road safety, vehicle performance, and human physical and cognitive performance (Guo et al. 2016).

The few studies that have assessed the relationship between extreme environmental temperatures and road traffic crashes have been undertaken in high-income countries, and the generalizability of their findings to low- and middle-income countries (LMICs) is limited due to variations in road safety regulations and compliance to them, climate adaptation policies, the quality of traffic infrastructure, and the age of the vehicle fleet, among other factors (Al-Hajj et al. 2022; Ameratunga et al. 2006; Heydari et al. 2019; Plummer and Boyle 2017). This limited generalizability is important given that much of the global burden of disease due to road traffic crashes including 90% of the world’s road traffic-related deaths takes place in LMICs (Naeem 2010). Moreover, these studies have focused on road traffic crashes in association with ambient temperatures; however, motor vehicle drivers have mainly been exposed to car indoor temperatures which could considerably differ from the environmental temperature due to, for example, the use of air conditioning systems. Such a difference between ambient and car indoor temperatures could result in considerable exposure misclassification. One way to avoid this limitation is to focus on road traffic crashes involving motorcyclists who are directly exposed to environmental temperatures. To date, no study has exclusively assessed the effects of extreme environmental temperatures on motorcyclist road traffic crashes. Accordingly, this study aimed to assess the association of exposure to extreme environmental temperatures (i.e., cold and hot) on motorcycle crashes in a city in Iran.

## Materials and methods

### Study setting

**Fig. 1:**
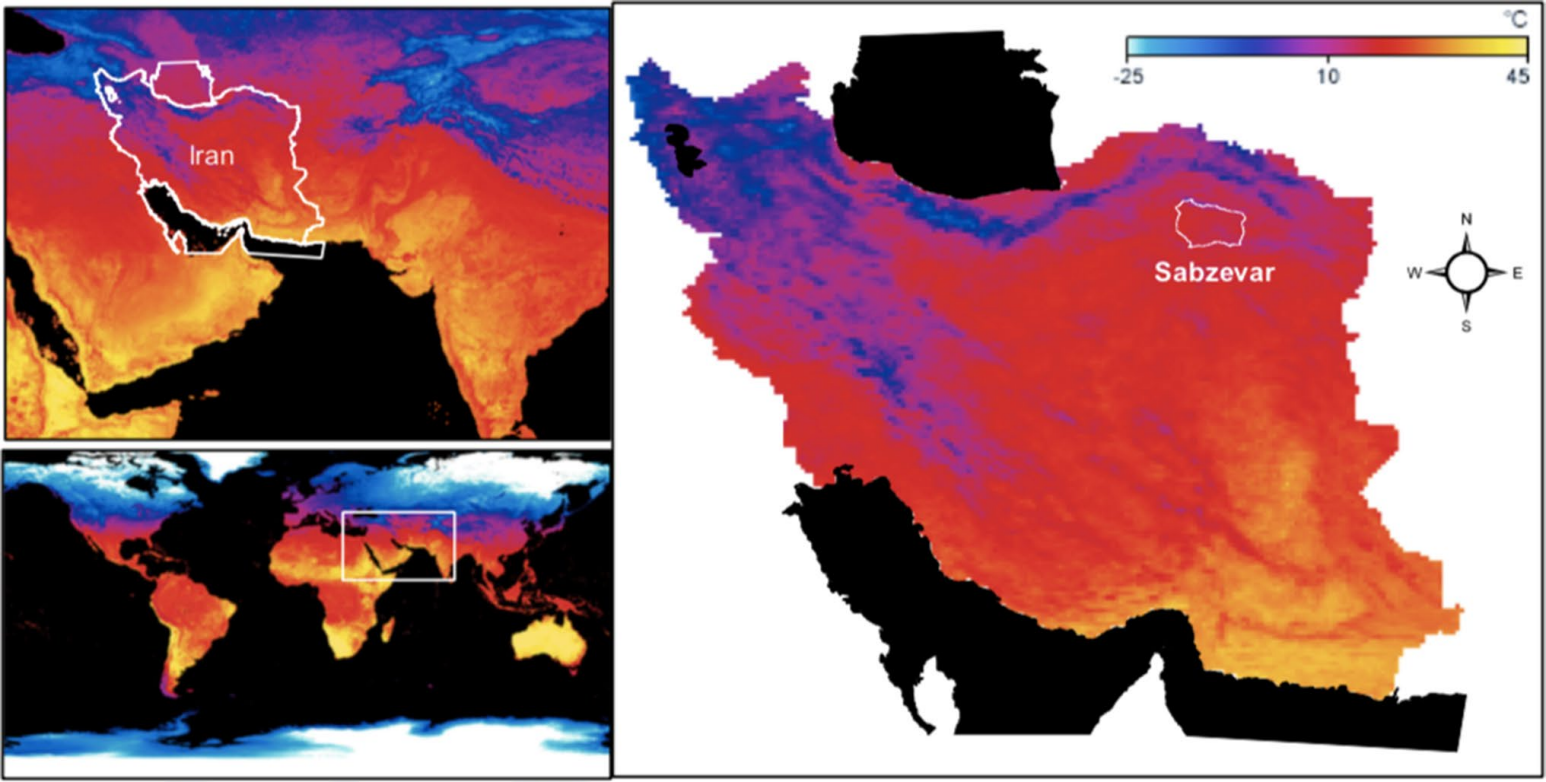
Location of the study area (Sabzevar City) in I.R. (Iran). The colors on the map represent the temperatures of the land surface as observed by MODIS in clear-sky conditions for August 2016. Yellow shows the warmest temperatures (up to 45 °C), and light blue shows the coldest temperatures (down to −25 °C). Black means “no data.” Raster file downloaded from: https://neo.sci.gsfc.nasa.gov/view.php?datasetId=MOD_LSTD_CLIM_M

The study was conducted in Sabzevar (coordinates: 36° 13′ N 57° 41′ E, elevation: 977 m), a city located in Khorasan Razavi Province in northeastern Iran (Fig. 1). Sabzevar has a population of 231,557 (2011 census) and a land area of 2676 km^2^, located at the edge of the Kavir Desert in the central plateau of Iran. It has an arid climate (according to the Köppen climate classification) with four distinct seasons (Peel et al. 2007). Khorasan Razavi Province has the highest rate of motorcycle crashes in Iran with an incidence rate of 55.8 per 10,000 residences (Gholamaliee et al. 2015). The study was approved by the Ethics Committee of the Shahid Sadoughi University of Medical Sciences (Ethics Committee approval number: IR.SSU.SPH.REC.1396.121).

### Data on medical attendance following motorcycle road traffic crash

Daily “medical attendance” was defined as the sum of hospital emergency department admission, or pre-hospital emergency system attendance registration due to motorcycle road traffic crashes. Data on daily counts of medical attendance in Sabzevar City as a result of motorcycle road traffic crash in the study period (between March 21, 2011, to June 30. 2017; hereafter as “study period”) were collected through two distinct sources: (i) emergency departments of all four hospitals of the city (three of them affiliated to the Sabzevar University of Medical Sciences and one affiliated to the public social security organization) and (ii) the pre-hospital emergency system. Since each victim can be approached by only one service, data from the above sources were complementary and completely distinct from each other. Direct admission to the hospital emergency department is mainly for those injured while riding a motorcycle within the city, whereas the pre-hospital emergency system provides service for motorcyclists injured in a road traffic crash occurring both within the city and in peri-urban areas, mainly Sabzevar-Neyshabur (up to 45 km from the city center) and Sabzevar-Shahrud (up to 60 km from the city center) roads. All records coded “V20–V29” (motorcycle rider injured in transport accident including collision with pedestrian or animal; collision with pedal cycle; collision with two- or three-wheeled motor vehicle; collision with car, pick-up truck, or van; collision with heavy transport vehicle or bus; collision with railway train or railway vehicle; collision with other non-motor vehicle; collision with fixed or stationary object; non-collision transport accident; other and unspecified transport accidents) according to the International Classification of Diseases version 10 (ICD-10-CM) were selected from the databases. Data registration was conducted at the time of admission to the hospital or contact with the pre-hospital emergency system and updated in the central system each day.

### Meteorological data

Meteorological data including daily minimum, mean and maximum temperature (°C), relative humidity (%), wind velocity (km/h), and precipitation (mm/day) were collected from the Sabzevar meteorological station (coordinates: 36° 12′ 27′′ N 57° 38′ 55′′ E) maintained by the Sabzevar Meteorological Organization.

As important indices to describe how hot the weather feels to people, Humidex and Wet Bulb Globe Temperature (WBGT) were computed using the following formula (Budd 2008; Masterton and Richardson 1979):

$$\mathrm{Humidex}=T+0.5555\times \left( vp-10\right)$$

$$\mathrm{WBGT}=0.567\ T\times 0.393\ vp\times 3.94$$

where *T* is the dry bulb temperature (°C) and *vp* is the vapor pressure (mbar for Humidex and mmHg for WBGT). Daily vapor pressure was calculated from relative humidity data and saturated vapor pressure at a given temperature using the method described by Finucane (Finucane 1998). Additional details on the calculation and definitions are shown in Table S1.

### Statistical analysis

We firstly used the distributed lag nonlinear model with quasi-Poisson regression to estimate the immediate and lagged associations of various temperature indicators (daily minimum, mean and maximum temperatures, Humidex, and WBGT) on medical attendance for motorcycle road traffic crashes. Then, we calculated the attributable fraction and attributable number of medical attendances for motorcycle crashes due to non-optimal temperatures. We further assessed the added effects of heat waves under various heat wave definitions on the medical attendances for motorcycle crashes.

In the distributed lag nonlinear model, we adjusted for the potential covariates, including wind velocity, precipitation, day of the week, relative humidity, holidays, and seasonal and long-term trends. In detail, day of the week and public holidays according to the Iranian calendar were included in the models as indicator variables. For the temperature-lag-health association and potential covariates presenting nonlinear effects, the natural cubic spline was applied and the generalized cross-validation (GCV) score was used to select the optimal *df* (Peng et al. 2006; Peng et al. 2017). Finally, the *df* of 3 was selected for precipitation and the wind velocity, and 7 *df* per year for time variable, 6 *df* was selected for temperature-health dimension, and 3 *df* for the lag dimension, with a maximum lag of 3 days, as a recent study on the impact of ambient temperature on occupational injuries showing effects up to 3 days (Martínez-Solanas et al. 2018). When assessing the effects of Humidex and WBGT, the relative humidity was not included in the model to avoid collinearity.

The 1st and 99th percentiles of thermal parameters were considered as “extremely cold” and “extremely hot” temperatures, respectively, while temperatures below the 25th percentile and higher than the 75th percentile were considered as “cold” and “hot” temperatures, respectively. Risk estimates (relative risk, RR) were calculated by centering the models at temperatures with minimum effect size according to the procedure described by Tobias et al. (Tobías et al. 2017). Furthermore, the RR over defined lag days for each day’s temperature was used to calculate the attributable fraction and attributable number of medical attendances for motorcycle crashes due to non-optimal temperatures using the following equations.

$${AF}_{x,t}=1-\exp \left(-\sum_{l=0}^L{\beta}_{x_{t-1},l}\right)$$

$${AN}_{x,t}={AF}_{x,t}\cdot {Y}_t$$

where the *AF*_*x,t*_ and *AN*_*x,t*_ are the attributable fraction and attributable number of medical attendances for motorcycle crashes at non-optimum temperature (*x*) at day *t*, respectively; *L* denotes the maximum lag of 3 days; *Y*_*t*_ is the observed number of medical attendances for motorcycle crashes at day t; and *β*_*x*_ denotes the log transformation of the RR of non-optimum temperature on medical attendances for motorcycle crashes, using temperature with minimum risk as a reference (Yang et al. 2015). Monte Carlo simulation was used to calculate the empirical confidence intervals (eCIs) of the attributable burden by generating 1000 replications (Gasparrini and Leone 2014; Greenland 2004).

### Added effect of heat wave

We applied sixteen different heat wave definitions according to Kent et al. (2014) (Table S2) to determine heat waves during our study period. We developed models similar to the main analyses to evaluate the added impact of heat waves on medical attendance for motorcycle crashes, separately for each heat wave definition.

### Sensitivity analyses

Given the fact Sabzevar is located in the vicinity of Kavir Desert (Dasht-e Kavir) and is often affected by desert dust storms, we conducted a sensitivity analysis by further adjustment of our analyses for visibility. Data on visibility was obtained from the Sabzevar meteorological station and had a 5-category ordinal scale (0: normal condition; 1: low level of dust; 2: medium level of dust; 3: visible distance less than 1000; 4: visible distance less than 200).

All time-series analyses were conducted using the “dlnm”, “splines,” and “mgcv” packages, in the R software (version 3.3.0) (Gasparrini 2011; Team 2013; Wood and Wood 2015).

## Results

In total, 36,079 medical attendances due to motorcycle road traffic crashes were recorded in Sabzevar City between March 21, 2011, to June 30, 2017 (average of 15.8 victims per day). All victims in this study were men. Assuming half of the Sabzevar population is an at-risk population (as only men ride motorcycles in Iran), the mean incidence rate of motorcycle road traffic crashes, based on our data, was 445 per 10,000 of the population. The minimum and maximum recorded temperatures in the study period were −11.2 and 45.4 °C, respectively. Figure S1 shows the distribution of selected parameters and thermal stress indices in this period.

Descriptions of the frequency of medical attendance for motorcycle road traffic crashes and meteorological parameters by year, month, day of the week, and holidays are shown in Table 1. An increasing trend (*p* < 0.001) was observed in the frequency of medical attendance during the study with the lowest frequency being observed in 2011 (average: 10.6; standard deviation: 2.8) and the highest frequency being observed in 2017 (25.5 ± 4.1). The highest and lowest frequencies of medical attendance for motorcycle road traffic crashes were observed in July and October, respectively. Sunday and Friday had respectively the highest (16.1 ± 6.2) and lowest (15.0 ± 5.8) frequencies of medical attendance for motorcycle road traffic crashes (in Iran, Friday is the weekend day and Saturday is the first working day of the week). The average frequency of medical attendance for motorcycle road traffic crashes in the working days (16.0 ± 6.0) was higher than that of holidays (15.2 ± 5.8) (*p* = 0.009) (Table 1).

**Table1:** Descriptive statistics on the daily number of motor vehicle crashes, temperature, relative humidity, wind, and precipitation (2011–2017)

	Daily number of crashes (mean ±SD)	Maximum temperature (mean ± SD)	Minimum temperature (mean ± SD)	Mean temperature (mean ± SD)	Maximum RH (mean ± SD)	Minimum RH (mean ± SD)	Mean RH (mean ± SD)	Wind velocity (mean ± SD)	Precipitation (mean ± SD)
Year									
2011	10.6 (2.8)	28.2 (10.7)	15.1 (9.0)	21.7 (9.8)	50.1 (24.6)	19.4 (17.6)	34.7 (20.1)	8.13 (2.4)	0.7 (2.9)
2012	13.2 (3.9)	24.1 (11.3)	11.4 (9.5)	17.8 (10.3)	55.1 (25.3)	24.0 (17.5)	39.5 (20.7)	7.5 (2.5)	0.5 (2.5)
2013	12.9 (4.0)	25.8 (10.6)	12.4 (9.4)	19.1 (9.9)	52.8 (23.1)	20.2 (14.0)	36.5 (17.7)	8.0 (2.6)	0.3 (1.8)
2014	13.3 (4.1)	25.0 (11.5)	12.0 (9.7)	18.5 (10.4)	53.2 (26.1)	21.1 (16.2)	37.1 (20.1)	11.8 (3.8)	0.5 (1.8)
2015	17.5 (4.1)	25.6 (10.8)	12.7 (9.1)	19.2 (9.9)	53.0 (23.8)	22.3 (15.5)	37.7 (19.1)	11.9 (3.4)	0.3 (1.4)
2016	21.5 (4.3)	26.0 (10.3)	12.7 (9.0)	19.4 (9.5)	53.5 (22.3)	18.6 (12.2)	36.1 (16.4)	11.7 (3.5)	0.4 (1.7)
2017	25.5 (4.1)	22.7 (10.9)	10.0 (9.1)	16.4 (9.9)	62.2 (21.9)	22.8 (17.4)	41.4 (19.5)	11.7 (3.7)	0.7 (2.8)
Month									
January	16.6 (5.5)	11.4 (4.3)	0.42 (3.4)	5.9 (3.5)	75.1 (15.3)	34.4 (13.5)	54.7 (13.2)	7.8 (2.9)	0.5 (1.7)
February	16.8 (6.1)	13.1 (5.7)	1.87 (4.6)	7.5 (4.8)	71.6 (16.5)	33.7 (16.5)	52.6 (15.6)	9.0 (3.4)	1.0 (3.2)
March	15.1 (5.7)	18.6 (5.2)	6.47 (4.4)	12.6 (4.5)	70.2 (16.6)	28.4 (13.4)	49.3 (13.2)	10.7 (3.9)	0.7 (2.6)
April	14.8 (6.3)	26.2 (5.0)	12.0 (3.9)	19.1 (4.2)	60.5 (19.5)	19.1 (11.8)	39.7 (14.7)	11.3 (4.1)	0.6 (2.1)
May	16.3 (6.4)	32.7 (3.6)	18.3 (3.1)	25.5 (3.2)	50.2 (19.1)	13.8 (8.0)	31.4 (12.8)	11.7 (3.9)	0.6 (2.5)
June	18.6 (6.2)	37.2 (3.0)	22.9 (2.9)	30.1 (2.7)	33.2 (14.1)	9.3 (5.4)	21.0 (9.1)	11.9 (3.5)	0.2 (1.8)
July	18.8 (4.8)	38.7 (2.6)	25.0 (2.4)	31.8 (2.3)	27.8 (10.2)	9.13 (4.0)	18.5 (6.6)	11.5 (3.3)	0.1 (0.5)
August	16.9 (4.4)	37.0 (2.7)	22.3 (2.6)	29.7 (2.5)	27.2 (10.4)	9.17 (5.0)	18.2 (7.2)	10.8 (3.0)	0.1 (0.3)
September	14.1 (5.5)	33.8 (3.2)	19.1 (2.6)	26.5 (2.7)	35.0 (13.5)	10.7 (5.5)	22.8 (8.9)	9.8 (2.8)	0.1 (1.0)
October	12.7 (4.8)	26.2 (4.9)	12.4 (3.8)	19.3 (4.2)	49.5 (17.8)	18.3 (11.0)	33.9 (13.6)	9.5 (3.2)	0.2 (1.1)
November	13.1 (5.5)	16.2 (5.3)	5.3 (3.8)	10.7 (4.2)	70.9 (18.3)	33.9 (18.0)	52.4 (17.1)	8.4 (3.5)	0.9 (3.1)
December	15.4 (5.6)	11.5 (4.2)	0.9 (3.5)	6.2 (3.6)	75.6 (16.7)	36.8 (16.6)	56.2 (15.6)	7.6 (2.9)	0.6 (2.5)
Day of week									
Saturday	16.0 (5.9)	25.4 (10.9)	12.5 (9.4)	19.0 (10.0)	53.1 (23.1)	21.1 (15.4)	37.0 (18.5)	9.9 (3.4)	0.4 (1.5)
Sunday	16.1 (6.2)	25.2 (11.2)	12.4 (9.4)	18.8 (10.2)	53.9 (24.5)	21.5 (16.2)	37.6 (19.6)	10.1 (3.7)	0.5 (1.9)
Monday	16.0 (5.8)	25.4 (11.2)	12.3 (9.5)	18.9 (10.2)	53.3 (24.5)	20.6 (15.7)	36.8 (19.1)	10.1 (3.9)	0.4 (2.0)
Tuesday	15.8 (5.9)	25.5 (11.1)	12.3 (9.4)	18.9 (10.1)	53.0 (24.1)	21.1 (16.2)	36.9 (19.4)	10.0 (3.7)	0.4 (1.9)
Wednesday	15.8 (5.8)	25.7 (11.0)	12.6 (9.4)	19.1 (10.1)	54.5 (24.8)	21.2 (16.1)	37.7 (19.6)	10.1 (3.9)	0.8 (3.2)
Thursday	15.7 (6.0)	25.5 (10.7)	12.6 (9.3)	19.1 (9.9)	54.6 (24.6)	21.1 (15.0)	37.7 (18.9)	10.1 (3.8)	0.3 (1.7)
Friday	15.0 (5.8)	25.5 (10.9)	12.5 (9.2)	19.0 (9.9)	53.9 (23.7)	21.3 (16.0)	37.5 (19.0)	10.2 (3.7)	0.4 (2.1)
Holidays									
No	16.0 (5.9)	25.6 (11.0)	12.5 (9.4)	19.0 (10.1)	53.4 (24.2)	21.1 (15.7)	37.1 (19.2)	10.0 (3.7)	0.5 (2.1)
Yes	15.2 (5.7)	25.1 (10.8)	12.1 (9.2)	18.6 (9.9)	55.0 (24.0)	21.4 (16.0)	38.1 (19.1)	10.2 (3.7)	0.5 (2.0)
Overall	15.8 (5.9)	25.5 (11.0)	12.4 (9.3)	19.0 (10.0)	53.7 (24.2)	21.1 (15.8)	37.3 (19.1)	10.1 (3.7)	0.5 (2.1)

Daily minimum, mean, and maximum temperature are based on °C; minimum, mean, and maximum relative humidity are based on %; wind velocity is based on kilometers per hour; and precipitation is based on millimeters per day

*RH*: Relative humidity

### Main analyses

**Fig. 2:**
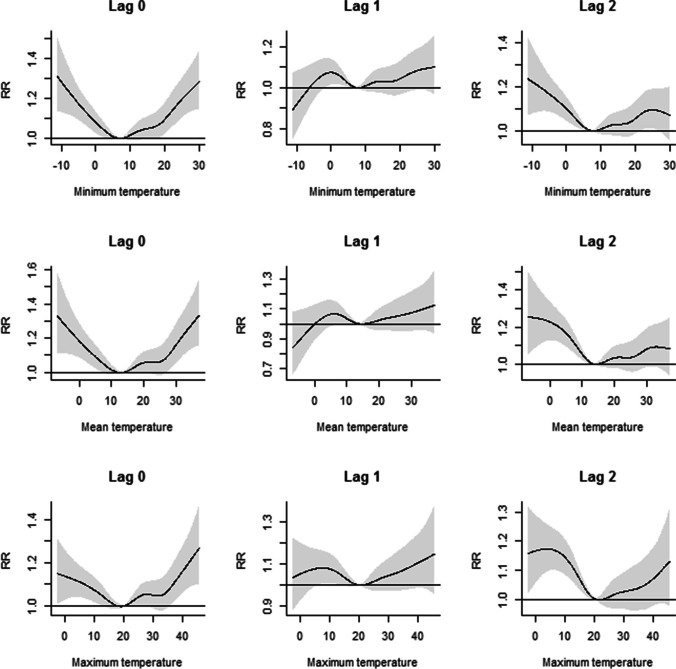
Dose-response relationship between selected thermal parameters and relative risk of medical attendance for motorcycle accidents at lag0, 1, 2. Results are based on a model adjusted for long-term trend, holidays, raining, and day of the week. All temperatures are based on degrees Celsius

The associations between minimum, mean, and maximum temperature and the daily frequency of medical attendance for motorcycle road traffic crashes at the 1st, 25th, 75th, and 99th percentiles of the thermal parameters over different lag days (up to three) are reported in Table 2. The “V”-shape association was found between minimum, mean, and maximum temperatures and the daily frequency of medical attendance for motorcycle road traffic crashes at lag 0 (Fig. 2). The lowest risk of medical attendance for motorcycle road traffic crashes was found at 7.6 °C (95% CI: −11.1, 8.7), 13.8 °C (95% CI: −2.4, 21.8), and 20.4 °C (95% CI: −11.5, 33.1) for minimum, mean, and maximum models, respectively (Fig. 2).

**Table 2:** The relative risk of medical attendance for motorcycle crash due to exposure to daily minimum, mean, and maximum temperature at 1st, 25th, 75th, and 99th percentile temperature distribution compared to the temperatures with minimum effect size (7.6, 13.8, and 20.5 °C for minimum, mean, and maximum temperature) at different lags

Lag effect	Extremely cold RR (95% CI)	Cold RR (95% CI)	Hot RR (95% CI)	Extremely hot RR (95% CI)
Minimum temperature				
Lag0	1.21 (1.13: 1.30)	1.02 (1.00: 1.04)	1.10 (1.03: 1.18)	1.24 (1.13: 1.35)
Lag1	1.02 (0.97: 1.07)	1.02 (1.01: 1.04)	1.04 (1.00: 1.09)	1.11 (1.04: 1.18)
Lag2	1.01 (0.96: 1.07)	1.02 (1.01: 1.04)	1.04 (0.99: 1.09)	1.06 (0.99: 1.13)
Lag3	1.20 (1.12: 1.29)	1.02 (1.00: 1.04)	1.09 (1.03: 1.17)	1.09 (1.00: 1.18)
Lag03	1.49 (1.40: 1.60)	1.09 (1.06: 1.11)	1.31 (1.20:1.43)	1.57 (1.40: 1.76)
Mean temperature				
Lag0	1.20 (1.11: 1.30)	1.02 (0.99: 1.05)	1.11 (1.03: 1.20)	1.27 (1.14: 1.41)
Lag1	1.04 (0.98: 1.10)	1.03 (1.01: 1.05)	1.05 (1.00: 1.12)	1.12 (1.04: 1.21)
Lag2	1.03 (0.97: 1.10)	1.03 (1.01: 1.05)	1.04 (0.98: 1.10)	1.06 (0.98: 1.14)
Lag3	1.18 (1.10: 1.28)	1.03 (1.01: 1.06**)**	1.07 (0.99: 1.15)	1.07 (0.96: 1.18)
Lag03	1.52 (1.42: 1.62)	1.12 (1.09: 1.15)	1.30 (1.19: 1.43)	1.60 (1.43: 1.79)
Maximum temperature				
Lag0	1.12 (1.05: 1.20)	1.02 (0.99: 1.04)	1.08 (1.01: 1.16)	1.20 (1.09: 1.32)
Lag1	1.09 (1.04: 1.15)	1.03 (1.01: 1.04)	1.06 (1.01: 1.12)	1.13 (1.06: 1.21)
Lag2	1.09 (1.03: 1.14)	1.03 (1.02: 1.05)	1.04 (0.99: 1.10)	1.08 (1.01: 1.15)
Lag3	1.10 (1.03: 1.18)	1.04 (1.01: 1.07)	1.02 (0.95: 1.09)	1.03 (0.94: 1.13)
Lag03	1.47 (1.37: 1.57)	1.12 (1.09: 1.16)	1.22 (1.12: 1.33)	1.51 (1.36: 1.68)

Extreme hot and hot conditions were calculated by comparing the 75th and 99th percentiles of the distribution. Extreme cold and cold conditions were also calculated using the 1st and 25th percentiles of the distribution. For minimum temperature: 1st percentile: −5.9 °C; 25th percentile: 4.2 °C; 50th percentile (median):12.9 °C; 75th percentile: 20.8 °C; 99th percentile: 28.3 °C. For maximum temperature: 1st percentile: 3.1 °C; 25th percentile: 15.7 °C; 50th percentile (median): 26.9 °C; 75th percentile: 35.5 °C; 99th percentile: 42.4 °C. For mean temperature: 1st percentile: −0.7 °C; 25th percentile: 9.9 °C; 50th percentile (median): 19.9 °C; 75th percentile: 28.2 °C; 99th percentile: 35.1 °C. The model included the following variables: minimum temperature, the long-time trend, day of the week, holidays, raining, wind velocity

*RR* relative risk, *CI* confidence interval

The associations were generally strongest at lag 0 and gradually weakened over lags 1 to 3 days. For all aforementioned indicators of thermal stress (minimum, mean, and maximum temperatures), we detected an increase in the risk of medical attendance for motorcycle road traffic crashes at extremely cold (1st percentile), hot (75th percentile), and extremely hot (99th percentile) temperatures at lag 0 (Table 2). For all three indicators, the highest risk increase was observed for extremely hot conditions (99th percentile), especially at lag 0. The increase in risk remained significant up to lag 1 for all models at extremely hot temperatures. Cumulative risks for lag 0 to lag 3 (lag03) showed a significantly increased risk for extremely cold, cold, hot, and extremely hot temperatures. The highest risk increases for lag03 observed for extremely hot temperatures, especially in the model with mean temperature (RR: 1.60; 95% CI: 1.43, 1.79). The lowest risk increases for lag 0 to lag 3 (lag03) were observed at cold temperatures for all the models.

Furthermore, 3590 (95% eCI: 2436, 4820), 3971 (95% eCI: 2851, 4998), and 3515 (95% eCI: 2492, 4688) medical attendances for motorcycle road traffic were attributable to the non-optimal daily minimum, mean, and maximum temperature, respectively, with the corresponding attributable fractions of 9.95% (95% eCI: 6.87, 13.24), 11.01% (95% eCI: 7.77, 14.06), and 9.74% (95% eCI: 6.8, 13.01) (Table 3).

**Table 3:** The attributable burden of medical attendance for motorcycle crashes due to multiple measures of thermal stress

Variable	Attributable number (*n*, 95% eCI)			Attributable fraction (%, 95% eCI)		
	Total	Cold	Heat	Total	Cold	Heat
Minimum temperature	3590 (2436, 4820)	1084 (749,1425)	2506 (1337,3609)	9.95 (6.87,13.24)	3.0 (2.09,3.9)	6.95 (3.84,10.18)
Mean temperature	3971 (2851,4998)	1343 (986,1676)	2628 (1535,3627)	11.01 (7.77,14.06)	3.72 (2.79,4.67)	7.29 (3.89,10.36)
Maximum temperature	3515 (2492,4688)	1317 (973,1697)	2197 (1070,3196)	9.74 (6.8,13.01)	3.65 (2.68,4.64)	6.09 (3.12,9.28)
Apparent temperature	4806 (534,8734)	0 (0,0)	4806 (745,8652)	13.32 (1.58,22.92)	0 (0,0)	13.32 (1.65,23.48)
Effective temperature	3921 (−76,7668)	0 (0,0)	3921 (195,7567)	10.87 (−0.24,21.23)	0 (0,0)	10.87 (0.14,21.28)
Net effective temperature	5135 (1046,8578)	0 (0,0)	5135 (1170,8405)	14.24 (2.37,24.28)	0 (0,0)	14.24 (3.74,23.71)
Humidex temperature	3635 (2596,4679)	1291 (957,1604)	2345 (1397,3321)	10.08 (7.03,12.98)	3.58 (2.68,4.5)	6.5 (3.63,9.05)
Wet bulb globe temperature	3463 (2427,4478)	1267 (934,1614)	2196 (1174,3131)	9.6 (6.74,12.58)	3.51 (2.6,4.41)	6.09 (3.41,8.56)

Cold was defined as a measure of thermal stress lower than the temperature with minimum effect, and heat as a measure of thermal stress higher than the temperature with minimum effect.

### Sensitivity analysis

The risk estimates after further adjustment of models for dust visibility (as a proxy of air pollution) were generally weaker compared to the main models (Tables S3). The estimated risks for the dust visibility–adjusted models with minimum, mean, and maximum temperature at the extremely cold (1st percentile) temperatures were attenuated to non-significant levels. For these models, the findings for hot (75th percentile) and extremely hot (99th percentile) conditions for minimum, mean, and maximum temperatures showed a uniform decreasing trend over the lags as observed in the main models. For extremely hot temperatures, the risk estimates at lag 0 remained significant after adjustment for visibility for minimum and mean temperatures (Tables S3).

### Further analysis

#### Heat waves

Depending on the definition of heat wave, between zero (definition: mean daily temperature > 99th percentile for ≥ 2 consecutive days) and 625 days (definition: maximum daily temperature > 35°C for ≥ 1 day) with heat wave were recorded between March 2011 and June 2017 (Table S2). The risk of medical attendance for motorcycle road traffic crashes in most of the heat wave definitions was significantly higher than non-heat wave days (Table S4). The highest relative risk of medical attendance for motorcycle crashes was observed in the conditions that the minimum daily temperature > 95th percentile for ≥ 2 consecutive days (RR: 1.12; 95% CI: 1.03, 1.23) and maximum daily apparent temperature > 90th percentile for ≥ 1 day (RR: 1.12; 95% CI, 1.03: 1.23). The cumulative risk of medical attendance for motorcycle crashes at the 3-day lag (lag 03) was also calculated for all definitions. The highest risk increases for medical attendance for motorcycle crashes at lag 03 were observed for conditions that the maximum daily temperature > 95th percentile for ≥ 2 consecutive days (RR: 1.41; 95% CI: 1.04,1.90).

#### Other measures of thermal stress

We observed an increased risk of medical attendance for motorcycle road traffic crashes associated with wet bulb globe temperature and Humidex, especially for extremely cold (1st percentile) and extremely hot (99th percentile) temperatures (Fig. S2; Table S5). Risk estimates for Humidex and WBGT index at extremely hot and extremely cold temperatures (99th percentile) were stronger compared to other thermal stress indicators (e.g., for extremely hot: RR: 1.26; 95% CI: 1.16, 1.38 and RR: 1.25; 95% CI: 1.15, 1.37 for Humidex and WBGT index, respectively).

## Discussion

This is the first study evaluating the impact of short-term exposure to hot and cold environmental temperatures and the risk of motorcycle crashes. This study, conducted in Iran, also adds to the paucity of evidence on the impact of extreme temperatures on road traffic crashes (Gariazzo et al. 2021; Xing et al. 2019), especially in LMICs where much of the burden of disease due to road traffic crashes occurs. We found an elevated risk of medical attendance for motorcycle crashes at both extremely cold (1st percentile) and hot (99th percentile) temperatures and also hot (75th percentile) temperatures. The associations were generally strongest at lag 0 and gradually weakened over lags 1 to 3 days. The risk estimates for extremely hot temperatures were generally larger than hot and extremely cold temperatures. These findings were generally consistent for different indicators of thermal stress. We also found an increased risk of medical attendance for motorcycle road traffic crashes during heat wave days (as defined in this study) compared to non-heat wave days.

### Interpretation of the findings

Our observed incidence rate was less than the reported incidence rate of 640 per 10,000 population for motorcycle road traffic crashes reported in a study undertaken in Tehran (Saadat and Soori 2011). To provide some comparisons, a Swedish study based on 16–25-year-old motorcyclists reported an incidence rate of 330 per 10,000 population (Zambon and Hasselberg 2006). We found that the highest frequency of medical attendance due to motorcycle road traffic crashes was during June and July and the lowest in October which was consistent with the observed higher frequency of crashes in the summer months and lowest frequency during the cold months in a previous Iranian study (Roudsari et al. 2004). However, a Korean study found no significant change in the risk of motorcycle crashes at different temperatures (Lee et al. 2014). Lower mobility (Moradi and Rahmani 2014) and shifting from motorcycle to public transportation and cars in the cold seasons (and vice versa for warm seasons) have been suggested as possible explanations for these seasonal variations (Parvareh et al. 2018).

Previous studies of the association between extreme temperature and road crashes were mainly focused on car drivers whose exposure to ambient temperature could have been modified by the use of air conditioning systems, among other factors. We observed an increased frequency of medical attendance for motorcycle road traffic crashes associated with extremely cold and extremely hot temperatures. These findings are in line with those of a study in Belgium reporting the increased frequency of road traffic crashes for two-wheelers (motorcyclists and cyclists) in warm and sunny weather (Masterton and Richardson 1979). In contrast to our findings, Nitschke et al. reported a decreased frequency of motorcycle road traffic crashes in temperatures above 35 °C in Adelaide, Australia (Nitschke et al. 2007). We are not aware of studies evaluating the associations of temperature over different lags on road traffic crashes; therefore, it is not possible to compare our findings for lags with those of previous studies. We observed suggestions for a decreasing trend in the strength of the associations from lag 0 to lag 3 days, which could suggest that the influence of thermal stress on motorcycle crashes is rather immediate.

We found a significant increase in the risk of medical attendance for motorcycle road traffic crashes during heat wave days compared to non-heat wave days for most of the applied definitions. Basagaña et al. reported that the risk of car crashes significantly increased by 2.9% (95% CI: 0.7%, 5.1%) during heat wave days (Basagaña et al. 2015). However, a study in Australia found a 33% decrease in road traffic crashes during heat waves (Liu et al. 2017). An analysis of road crashes in the UK showed that most crashes occurred on days with fine weather, although no definition of “fine weather” was provided (im Kampe et al. 2016). Such heterogeneity in findings could be due to differences defining the heat waves, climate, cultural contexts, and use of air conditioners among other factors.

We found that the points with the lowest effect size for all temperature indices and parameters used in this study located at cold temperatures (between the 25th and 50th percentiles) part of the distributions. Short-term cooling in a simulated driving task was associated with lower self-reported sleepiness, eyelid closures, and driving errors (Schmidt et al. 2017). The study recommended cooling to 17 °C as a preventive measure against cognitive fatigue during driving (Schmidt et al. 2017). However, another study reported an increase in risky driving behavior in moderately cold temperatures with the absence of hypothermia (Morris and Pilcher 2016).

### Potential mechanisms

Weather characteristics can influence road safety, vehicle performance, and also a driver’s behavior and abilities (Theofilatos and Yannis 2014). Vehicle performance could be influenced by weather characteristics. Brake pads’ performance could be affected by high temperatures due to brake fade and by cold temperatures because of the increase in the risk of brake sticking (Krasnoshtanov et al. 2018). Moreover, temperatures could influence human behavior and cognitive performance (Taylor et al. 2016). In an experimental study, driving performance at cold (5 °C; 50% relative humidity) and warm (35 °C; 50% relative humidity) ambient temperatures decreased by 16% and 13%, respectively (Daanen et al. 2003). Another study showed that under extreme heat conditions (outdoor temperature of 34 °C), performance losses could reach 75% for demanding tasks such as correctly identifying a signal and reacting in due time (Lenzuni et al. 2014). Executive function and manual dexterity could also be negatively affected especially in cold environments (Heus et al. 1995). Shivering in extremely cold conditions could decrease the performance of the motorcyclists (Daanen et al. 2003). A meta-analysis of the available studies on the effect of thermal stress on mental performance in humans reported comparable effect sizes for heat and cold stress (Hancock et al. 2007). It showed that thermal stress could influence psychomotor and perceptual task performance and, to a lesser extent, cognitive performance (Hancock et al. 2007). This review suggested that thermal stress could force the motorcyclists to direct their attention to cope with the stress, which in turn could reduce the capacity to process task-relevant information (Hancock et al. 2007). In addition, dehydration due to prolonged exposure to elevated temperature hot days can also increase the frequency of the driver’s error (Watson et al. 2015).

Extreme temperatures, especially hot temperatures, could also induce a sense of tiredness, anxiety, and aggression that in turn could increase the possibility of committing risky behaviors and hence result in road traffic crashes (Manning and Clayton 2018). Accordingly, an indirect pathway underlying our findings could be the willingness of motorcyclists to reach sooner to the indoor environments in the case of hot or cold temperatures, which by itself could increase the risk of their violation of speed limits and therefore enhance the chance of crashes. Liang et al. found that the speed of passenger cars was higher in hot temperatures compared to the freezing temperatures (Liang et al. 1998). Another possible indirect pathway for the association of ambient temperature extremes and motorcycle crashes is the effect of temperature on sleep quality and sleep disturbance in the nights preceding the crash. Falling asleep during driving has been reported to be more frequent in summer than in winter (Radun and Radun 2006).

### Strengths and limitations

A key strength of this study is the setting within a low- and middle-income country, where road traffic crashes contribute a high burden of morbidity, and there is not a lot of available literature on extreme temperatures and health. Given that in Iran women do not ride motorcycles, all of the motorcyclists included in our analyses were male, which, on the one hand, made our sample more homogeneous and on the other hand could limit the comparability of our findings with those of other studies in other countries. Using readings from one meteorological station for assessing the exposure of urban participants to temperature could have resulted in exposure misclassification. The role of distance with the meteorological station for different participants across the city and also heat islands cannot be neglected. Sabzevar city is relatively small with homogenous traffic building texture; therefore, we did not expect a considerable exposure misclassification due to the use of data from a single monitoring station; however, our study could have benefited from integrating data on the location of crashes to characterize the condition of the roads, and inclusion of a high-resolution heat map over the study area including temperature hot spots. We did not have access to data on the location of crashes and therefore were not able to implement this idea in our study. Similarly, we did not have access to daily traffic volume data in our study area to account for it in our analyses. We tried to address the effect of traffic volume in our analysis by using the day of week and holidays in the models (Bergel-Hayat et al. 2013). Moreover, we did not have data on traveling time before the crash. Longer traveling time could result in both longer at-risk time and more cumulative exposure.

## Conclusions

We observed an increased frequency of medical attendance for motorcycle crashes in association with short-term exposure to hot and extreme hot and cold temperatures. The ongoing changes in the climate are expected to increase the frequency, intensity, and duration of extreme weather conditions. In this context, our findings, if confirmed by future studies, could have important implications for policymakers, given the considerable burden associated with road traffic crashes, particularly in LMICs. Road traffic crashes are projected to be the fifth leading cause of mortality by the year 2030, mainly because of economic growth and hence greater access to varying modes of mobility in LMICs (Mathers and Loncar 2006). It is estimated that road traffic crashes are responsible for approximately 3% of the loss of gross domestic product (GDP) in LMICs (WHO 2015). In Iran, it was estimated that the economic burden of road traffic injuries on only the health sector accounted for 2.18% of the country’s total GDP in 2011 (Behnood et al. 2017). There is therefore a need to replicate our findings in other settings, especially in LMICs, with different climates, traffic cultures, and fleet, while applying a more elaborated heat map. Additionally, if these findings can be replicated in cities with extremes in temperature and concur with the current finding, then, public health messaging needs to be targeted towards these climate events to mitigate the increased risk of motorcyclists during these periods.

## Supplementary information

ESM 1 (PDF 660 kb)

## Data Availability

All the data, codes, and analyses for this manuscript are available upon request from MJZS and PD.
