# Supplementary material for: Extreme environmental temperatures and motorcycle crashes: a time-series analysis
Source: Environ Sci Pollut Res Int. 2022 Jun 6;29(50):76251–62. doi: 10.1007/s11356-022-21151-8 (PMC9553821; doi:10.1007/s11356-022-21151-8)
Supplement: Supplementary file 1 — (PDF 660 kb) [file 11356_2022_21151_MOESM1_ESM.pdf]

Supplemental Materials

Extreme environmental temperatures and motorcycle crashes: A time-series analysis

Mohammad Javad Zare Sakhvidi[1], Jun Yang[2], Danial Mohammadi[1], Hussein FallahZadeh[3], Amirhooshang Mehrparvar[4], Mark Stevenson[5], Xavier Basagaña[6,7,8], Antonio Gasparrini[9,10,11], Payam Dadvand[6,7,8*]

1-Department of Occupational Health, School of Public Health, Shahid Sadoughi University of Medical Sciences, Yazd, Iran

2-School of Public Health, Guangzhou Medical University, Guangzhou, 511436, China

3-Department of Epidemiology and Biostatistics, School of Public Health, Shahid Sadoughi University of Medical Sciences, Yazd, Iran

4-Department of Occupational Medicine, Faculty of Medicine, Shahid Sadoughi University of Medical Sciences, Yazd, Iran

5- Melbourne School of Design/Melbourne School of Population and Global Health, The University of Melbourne, Melbourne, Victoria, Australia

6-ISGlobal, Barcelona, Spain.

7-Universitat Pompeu Fabra (UPF), Barcelona, Spain.

8-CIBER Epidemiología y Salud Pública (CIBERESP), Spain

9-Department of Public Health, Environments and Society, London School Hygiene & Tropical Medicine, London, UK.

10- Centre for Statistical Methodology, London School Hygiene & Tropical Medicine, London, UK

11- Centre on Climate Change and Planetary Health, London School of Hygiene & Tropical Medicine

Table of contents

Title	Page
**Table S1.** Definitions of the temperature and indices which are used in the study.	24
**Table S2.** The number of "heat wave" days during 2011-2017 (based on different heat wave definitions borrowed from Kent et.al.; 2014), and the corresponding number of daily medical attendance for motorcycle accidents (mean ± SD) at each heat wave definition.	25
**Table S3.** The relative risk of medical attendance for motorcycle crashes due to exposure to the daily minimum temperature at 1st, 25th, 75th, and 99th percentile of minimum temperature compared to the temperature with minimum effect with models additionally adjusted for dust visibility.	26
**Table S4.** The relative risk of daily medical attendance for motorcycle accidents according to different heat wave definitions.	27
**Table S5.** The relative risk of medical attendance for motorcycle collision due to exposure to daily Humidex, and Wet Bulb Globe Temperature (WBGT) at 1st, 25th, 75th, and 99th percentile of indices compared to the value with the lowest effect size (12.1, and 14.5 °C for Humidex and WBGT respectively).	28
**Figure S1.** Distribution of thermal and meteorological parameters in the study period (March 2011 to June 2017) based on the readings from the nearest station to Sabzevar city.	29
**Figure S2.** Dose-response relationship between Humidex and wet bulb globe temperature (WBGT) and relative risk of medical attendance for motorcycle accidents at lag 0, 1, 2 days. Results are based on a model adjusted for long-term trends, holidays, rain, and day of the week.	30

Table S1. Definitions of the temperature and indices which are used in the study.

Parameter	Definition	Calculation
Maximum Temperature (°C)	The maximum recorded temperature during 24 hrs.	--
Minimum Temperature (°C)	The minimum recorded temperature during 24 hrs.	--
Mean Temperature (°C)	The average of recorded temperature during 24 hrs.	Average of all hourly recorded temperature during 24 hrs.
Apparent temperature (°C)	The equivalent temperature is perceived by humans, by the combined effects of air temperature, relative humidity, and wind speed (Nguyen et al. 2014).	$AT = T + 0.33\ vp - 0.7\ v - 4$
Effective temperature (°C)	The temperature in an environment with 50% relative humidity in which a hypothetical person experiences the same amount of heat losses as in the situation under analysis (Sala-Lizarraga et al. 2019).	$T - 0.4 \times (T - 10) \times (1 - (RH/100))$
Net effective temperature (°C)	Net effective temperature (NET) integrates temperature, relative humidity, and wind speed as a cooling indicator (Hentschel 1986).	$37 - \frac{37 - T}{0.68 - 0.0014RH + \frac{1}{1.76 + 1.4V^{0.75}}} - 0.9T \times (1 - \frac{RH}{100})$
Humidex temperature (°C)*	perceived thermal feeling of a person, by combining the effect of heat and humidity (Rana et al. 2013).	$T + 0.5555 \times (vp - 10)$
Wet bulb globe temperature (°C)	The measure of the heat stress in direct sunlight, takes into account: temperature, humidity, and wind speed (Budd 2008, Zhao et al. 2015).	$0.567\ T \times 0.393\ vp \times 3.94$

T: dry globe temperature (°C); vp: vapor pressure (Pa; for Humidex mbar; for WBGT as hPa); RH: relative humidity (%); v: wind velocity (m/s)
*: Humidex can also be regarded as a unitless index.

References:

Budd, G. M. (2008). Wet-bulb globe temperature (WBGT)—its history and its limitations. *Journal of Science and Medicine in Sport*, *11*(1), 20-32.

Hentschel, G. (1986). A human biometeorology classification of climate for large and local scales. In *Proc. WMO/HMO/UNEP Symposium on Climate and Human Health* (pp. 22-26).

Nguyen, J. L., Schwartz, J., & Dockery, D. W. (2014). The relationship between indoor and outdoor temperature, apparent temperature, relative humidity, and absolute humidity. *Indoor air*, *24*(1), 103-112.

Rana, R., Kusy, B., Jurdak, R., Wall, J., & Hu, W. (2013). Feasibility analysis of using humidex as an indoor thermal comfort predictor. *Energy and Buildings*, *64*, 17-25.

Sala-Lizarraga, J. M., & Perez, A. P. (2019). *Exergy Analysis and Thermoeconomics of Buildings: Design and Analysis for Sustainable Energy Systems*. Butterworth-Heinemann.

Zhao, Y., Ducharne, A., Sultan, B., Braconnot, P., & Vautard, R. (2015). Estimating heat stress from climate-based indicators: present-day biases and future spreads in the CMIP5 global climate model ensemble. *Environmental Research Letters*, *10*(8), 084013.

Table S2. The number of "heat wave" days during 2011-2017 (based on different heat wave definitions borrowed from Kent et.al.; 2014), and the corresponding number of daily medical attendance for motorcycle accidents (mean ± SD) at each heat wave definition.

Code	Heat wave definition	Number of days in the definition	Number of daily medical attendance for motorcycle accidents in definition (Mean ± SD)
H01	Mean daily temperature > 95th percentile for ≥ 2 consecutive days	19	19.21 ± 4.26
H02	Mean daily temperature > 90th percentile for ≥ 2 consecutive days	74	19.79 ± 5.30
H03	Mean daily temperature > 98th percentile for ≥ 2 consecutive days	4	15.25 ± 2.87
H04	Mean daily temperature > 99th percentile for ≥ 2 consecutive days	0	-
H05	Minimum daily temperature > 95th percentile for ≥ 2 consecutive days	12	19.83 ± 4.87
H06	Maximum daily temperature > 95th percentile for ≥ 2 consecutive days	4	21.75 ± 5.85
H07	Maximum daily temperature ≥ 81st percentile every day, ≥ 97.5th percentile for ≥ 3 nonconsecutive days, and consecutive day average ≥ 97.5th percentile	4	15.25 ± 2.87
H08	Maximum daily apparent temperature > 85th percentile for ≥ 1 day	16	19.75 ± 4.41
H09	Maximum daily apparent temperature > 90th percentile for ≥ 1 day	11	18.90 ± 5.10
H10	Maximum daily apparent temperature > 95th percentile for ≥ 1 day	4	15.25 ± 2.87
H11	Maximum daily temperature > 35°C (95°F) for ≥ 1 day	625	17.90 ± 5.57
H12	Minimum daily temperature > 26.7°C (80.1°F) or maximum daily temperature > 40.6°C (105.1°F) for ≥ 2 consecutive days	37	20.83 ± 5.59
H13	Maximum daily heat index* > 80°F for ≥ 1 day	84	19.92 ± 5.32
H14	Maximum daily heat index > 90°F for ≥ 1 day	60	20.90 ± 5.08
H15	Maximum daily heat index > 105°F for ≥ 1 day	19	19.73 ± 5.76
H16	Maximum daily heat index > 130°F for ≥ 1 day	12	19.91 ± 5.17

* Heat index is a function of air temperature and humidity, parameterized to take account of other environmental factors.
Reference: Kent, Shia T., et al. "Heat waves and health outcomes in Alabama (USA): the importance of heat wave definition." Environmental health perspectives 122.2 (2014): 151.

Table S3. The relative risk of medical attendance for motorcycle crashes due to exposure to the daily minimum temperature at 1st, 25th, 75th, and 99th percentile of minimum temperature compared to the temperature with minimum effect with models additionally adjusted for dust visibility.

Lag effect	Extremely cold	Cold	Hot	Extremely hot
Minimum temperature				
Lag0	1.05 (1.00: 1.11)	1.01 (0.99: 1.02)	1.05 (0.99: 1.10)	**1.08 (1.01: 1.16)**
Lag1	1.01 (0.97: 1.05)	**1.02 (1.01: 1.03)**	1.01 (0.98: 1.05)	1.05 (1.00: 1.10)
Lag2	1.01 (0.97: 1.06)	**1.02 (1.01: 1.03)**	1.02 (0.98: 1.05)	1.04 (0.99: 1.09)
Lag3	**1.07 (1.01: 1.13)**	1.01 (0.99: 1.02)	1.06 (1.00: 1.11)	1.05 (0.98: 1.12)
Mean temperature				
Lag0	1.05 (0.99: 1.12)	1.01 (0.99: 1.03)	1.06 (1.00: 1.13)	**1.10 (1.01: 1.19)**
Lag1	1.03 (0.98: 1.08)	**1.02 (1.01: 1.04)**	1.02 (0.97: 1.06)	1.05 (0.99: 1.11)
Lag2	1.03 (0.98: 1.08)	**1.02 (1.01: 1.04)**	1.01 (0.97: 1.06)	1.03 (0.97: 1.09)
Lag3	1.04 (0.98: 1.11)	1.01 (0.99: 1.04)	1.05 (0.99: 1.11)	1.04 (0.96: 1.12)
Maximum temperature				
Lag0	1.04 (0.98: 1.09)	1.01 (0.99: 1.03)	1.05 (0.99: 1.11)	1.07 (0.99: 1.15)
Lag1	**1.04 (1.01: 1.08)**	1.01 (1.00: 1.03)	1.03 (0.99: 1.08)	1.06 (1.00: 1.12)
Lag2	1.04 (1.00: 1.08)	**1.02 (1.01: 1.03)**	1.02 (0.98: 1.06)	1.04 (0.98: 1.09)
Lag3	1.01 (0.96: 1.07)	**1.03 (1.01: 1.05)**	1.01 (0.96: 1.07)	1.00 (0.93: 1.08)

RR: Relative risk, CI: Confidence interval.
Extreme hot and hot conditions were calculated by comparing the 75th and 99th percentile of the distribution. Extreme cold and cold conditions were also calculated using the 1st and 25th percentile of the distribution.
For minimum temperature: 1st percentile: -5.9 °C; 25th percentile: 4.2 °C; 50th percentile (median):12.9 °C; 75th percentile: 20.8 °C; 99th percentile: 28.3 °C.
For maximum temperature: 1st percentile: 3.1 °C; 25th percentile: 15.7 °C; 50th percentile (median):26.9 °C; 75th percentile: 35.5 °C; 99th percentile: 42.4 °C.
For mean temperature: 1st percentile: -0.7 °C; 25th percentile: 9.9 °C; 50th percentile (median):19.9 °C; 75th percentile: 28.2 °C; 99th percentile: 35.1 °C.
The model included the following variables: minimum temperature, the long-time trend, day of the week, holidays, raining, wind velocity, visibility

Table S4. The relative risk of daily medical attendance for motorcycle accidents according to different heat wave definitions.

Heat wave definition*	Heat wave day (yes/no)	Accident frequency		Lag specific relative risk**	
		Mean	Standard Deviation	Lag 0	Lag 03
H01	No	15.76	5.92	Reference	Reference
	Yes	19.21	4.26	**1.08 (1.01: 1.16)**	1.13 (1.00: 1.28)
H02	No	15.66	5.89	Reference	Reference
	Yes	19.80	5.30	**1.07(1.03: 1.11)**	**1.16(1.09: 1.23)**
H03	No	15.79	5.92	Reference	Reference
	Yes	15.25	2.87	0.99(0.84: 1.16)	1.10(0.82: 1.48)
H04	No	15.78	5.91	NA	NA
	Yes	--	--	NA	NA
H05	No	15.77	5.91	Reference	Reference
	Yes	19.83	4.87	**1.12(1.03: 1.23)**	**1.24(1.07: 1.44)**
H06	No	15.78	5.91	Reference	Reference
	Yes	21.75	5.85	1.09 (0.95: 1.26)	**1.41(1.04: 1.90)**
H07	No	15.79	5.92	Reference	Reference
	Yes	15.25	2.87	0.99(0.84: 1.16)	1.10(0.82: 1.48)
H08	No	15.76	5.92	Reference	Reference
	Yes	19.75	4.41	1.08(1.00: 1.16)	1.12 (0.98: 1.28)
H09	No	15.78	5.91	Reference	Reference
	Yes	18.91	5.10	**1.12 (1.03: 1.23)**	1.18 (1.00: 1.40)
H10	No	15.79	5.92	Reference	Reference
	Yes	15.25	2.87	0.99(0.84: 1.16)	1.10(0.82: 1.48)
H11	No	15.00	5.85	Reference	Reference
	Yes	17.91	5.57	**1.05(1.02: 1.08)**	**1.15(1.10: 1.20)**
H12	No	15.71	5.88	Reference	Reference
	Yes	20.84	5.59	**1.09(1.03: 1.14)**	**1.12(1.03: 1.21)**
H13	No	15.63	5.88	Reference	Reference
	Yes	19.93	5.32	**1.06(1.02: 1.10)**	**1.18(1.11: 1.26)**
H14	No	15.65	5.87	Reference	Reference
	Yes	20.90	5.08	**1.08(1.03: 1.12)**	**1.18(1.10: 1.27)**
H15	No	15.76	5.92	Reference	Reference
	Yes	19.74	5.76	1.07 (1.00: 1.15)	**1.23 (1.06: 1.42)**
H16	No	15.77	5.91	Reference	Reference
	Yes	19.92	5.17	**1.11(1.01: 1.21)**	**1.34 (1.11: 1.63)**

*: for detailed information on heat waves refer to table S2.
** Models are adjusted for relative humidity, rain, wind velocity, day of the week, long-term trend, and holidays.

Table S5. The relative risk of medical attendance for motorcycle collision due to exposure to daily Humidex, and Wet Bulb Globe Temperature (WBGT) at 1st, 25th, 75th, and 99th percentile of indices compared to the value with lowest effect size (12.1, and 14.5 °C for Humidex and WBGT respectively).

**Lag effect**	**Extremely cold**	**Cold**	**Hot**	**Extremely hot**
Humidex				
Lag0	**1.23 (1.14: 1.33)**	1.02 (1.00: 1.04)	**1.12 (1.04: 1.20)**	**1.26 (1.16: 1.38)**
Lag1	1.02 (0.96: 1.08)	**1.03 (1.01: 1.05)**	1.03 (0.98: 1.09)	**1.08 (1.01: 1.15)**
Lag2	1.01 (0.95: 1.07)	**1.03 (1.01: 1.05)**	1.02 (0.97: 1.07)	1.02 (0.95: 1.08)
Lag3	**1.20 (1.11: 1.29)**	**1.03 (1.01: 1.05)**	1.06 (0.99: 1.14)	1.05 (0.96: 1.15)
WBGT (Wet Bulb Globe Temperature)				
Lag0	**1.24 (1.14: 1.34)**	1.02 (1.00: 1.05)	**1.12 (1.04: 1.20)**	**1.25 (1.15: 1.37)**
Lag1	1.02 (0.96: 1.08)	**1.03 (1.01: 1.05)**	1.03 (0.98: 1.08)	1.07 (1.00: 1.13)
Lag2	1.01 (0.95: 1.07)	**1.03 (1.01: 1.05)**	1.01 (0.96: 1.06)	1.01 (0.95: 1.07)
Lag3	**1.20 (1.11: 1.29)**	1.03 (1.00: 1.05)	1.07 (1.00: 1.14)	1.06 (0.97: 1.15)

RR: Relative risk, CI: Confidence interval.
Extreme hot and hot conditions were calculated by comparing the 75th and 99th percentile of the distribution. Extreme cold and cold conditions were also calculated using the 1st and 25th percentile of the distribution.
For Humidex: 1st percentile: -4.6 °C; 25th percentile: 8 °C; 50th percentile (median):18.6 °C; 75th percentile: 27 °C; 99th percentile: 35.6 °C.
For WBGT: 1st percentile: -16.1 °C; 25th percentile: -1.5 °C; 50th percentile (median):10.2 °C; 75th percentile: 18.8 °C; 99th percentile: 26.1 °C.
The model included the following variables: apparent temperature, the long-time trend, day of the week, holidays, rain,

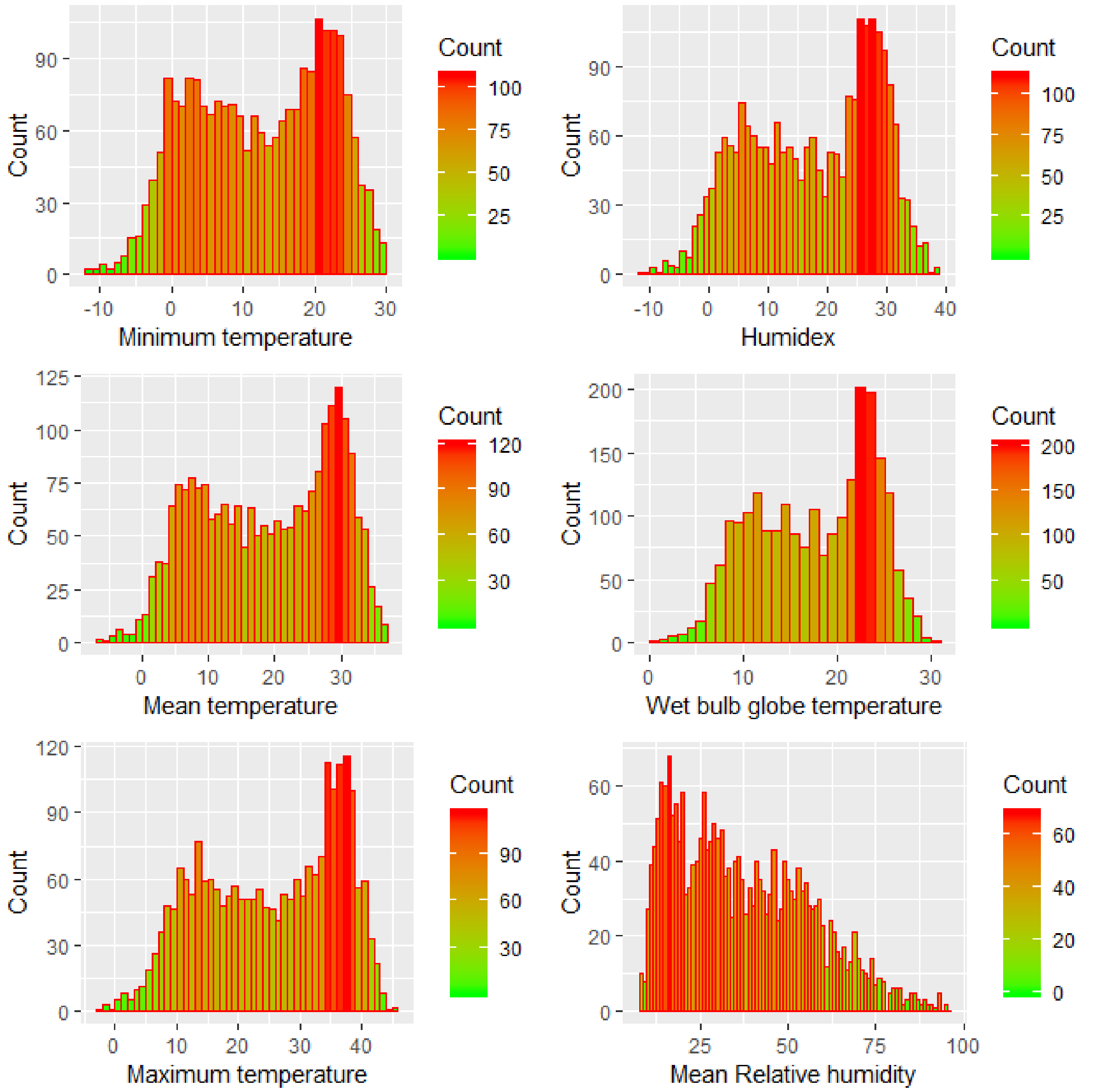

Figure S1. Distribution of thermal and meteorological parameters in the study period (March 2011 to June 2017) based on the readings from the nearest station to Sabzevar city.

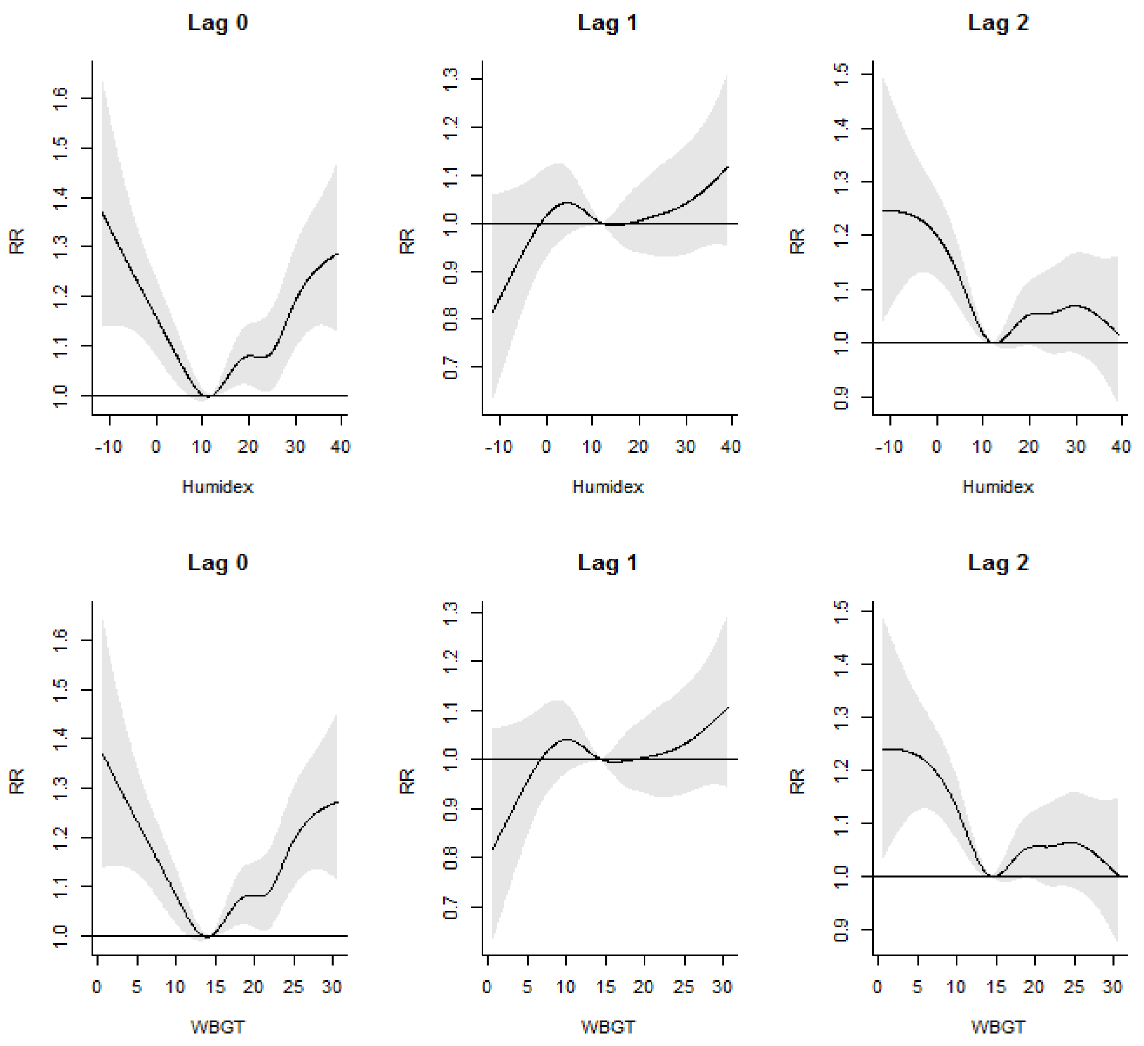

Figure S2. Dose-response relationship between Humidex (°C) and wet bulb globe temperature (WBGT; °C) and relative risk of medical attendance for motorcycle accidents at lag 0, 1, 2 days. Results are based on a model adjusted for long-term trends, holidays, rain, and day of the week.
